# The Validity and Reliability of the Malay Version of the Cyberbullying Scale among Secondary School Adolescents in Malaysia

**DOI:** 10.3390/ijerph182111669

**Published:** 2021-11-06

**Authors:** Zaitun Mohd Saman, Ab Hamid Siti-Azrin, Azizah Othman, Yee Cheng Kueh

**Affiliations:** 1 Clinical Research Centre (CRC), Hospital Pakar Sultanah Fatimah, Muar 84000, Johor, Malaysia; zaitunms@gmail.com; 2 Biostatistics and Research Methodology Unit, School of Medical Sciences, Universiti Sains Malaysia, Kubang Kerian 16150, Kelantan, Malaysia; 3 Department of Paediatrics, School of Medical Sciences, Universiti Sains Malaysia, Kubang Kerian 16150, Kelantan, Malaysia; azeezah@usm.my

**Keywords:** online, cyber victim, student, internet, adolescents, confirmatory factor analysis

## Abstract

The effect of cyberbullying among adolescents in Malaysia is not much studied. The Cyberbullying Scale (CBS) has been validated to be used among English speaking adolescents to measure cyberbullying but not in Malay language. Therefore, its validity should be established before use in the Malaysian context. Thus, the study aimed to evaluate the validity and reliability of the Malay version of the CBS (CBS-M) among secondary school students. The study was cross-sectional and involved a self-administered questionnaire with 16 items from CBS-M, and 21 items from the Depression, Anxiety, and Stress Scale (DASS-21). Participants were recruited using a multi-stage sampling method. The validity of the CBS-M was tested in two phases, namely, exploratory factor analysis (EFA) and confirmatory factor analysis (CFA). Spearman’s correlation was used to examine the strength of the relationship between the CBS and subscales from DASS-21 to further support the validity of CBS-M. A total of 401 respondents from Muar, Johor, participated. The mean age was 14.6 years (SD = 1.25). EFA results indicated a one-factor model of CBS-M with a total variance extracted of 33.9%. Internal consistency measured by Cronbach’s alpha reached 0.87. The model was then tested using CFA. The initial model did not fit the data well. Thus, several model re-specifications were conducted on the initial model. The final measurement model of CBS-M fit the data well with acceptable fit indices (CFI = 0.946, TLI = 0.932, SRMR = 0.055, RMSEA = 0.049). The composite reliability for CBS-M was satisfactory with a value of 0.832. The CBS-M questionnaire is a valid and reliable tool for measuring cyberbullying among young adolescents in Malaysia.

## 1. Introduction

In the modern era, the Internet is accessible to nearly everyone. The number of Internet users worldwide for 2019 reached 4.13 billion, which indicated an increase from 3.92 billion in 2018 [1]. In January 2020, Internet World Stats [2] recorded a total of 4.57 billion Internet users worldwide. Similarly, Internet users are increasing in Malaysia. The Malaysian Communications and Multimedia Commission [3] noted increases of 76.9% and 87.4% in 2016 and 2018, respectively. Furthermore, Mohd Isa et al. [4] indicated that 70% of Internet users started using the Internet since primary school age or below 12 years old. Approximately 37% of the Malaysian youth reported using the Internet for 6 to 12 h daily (moderate users) and 17% used the Internet for 12 to 18 h daily (at-risk users) [5].

The potential risks of Internet use include visiting pornographic websites, unwanted sexual content, violence in online video games, Internet addiction, and cyberbullying [6,7,8]. Balakrishnan [9] conducted a study among Malaysian aged between 17 to 35 years old, reported that 39.7% of users admitted to being cyberbullied online whereas 33.6% stated that they had cyberbullied anyone. However, among the youth in Malaysia, 62.3% reported being victims of cyberbullying [10]. But still, cases of cyberbullying in Malaysia may be underreported due to lack of cyberbullying tools in Malay language.

Cyberbullying is an aggressive, intentional act conducted by an individual or a group using electronic forms of contact against a victim with difficulty in defending himself or herself [11]. According to Hasebrink et al. cyberbullying is defined as bullying on the Internet or mobile phones, and online bullying as bullying on the Internet only” [12]. Cyberbullying is especially harmful to children because it can lead to social anxiety, depression, and loneliness [13,14]. Additionally, a significant association was observed between experiences of cyberbullying and low academic achievement [15]. Suicidal ideation and attempt were reported to be significantly associated with cyberbullying [16]. Additionally, the Institute for Youth Research Malaysia [10] reported that victims of cyberbullying became emotionally unstable (25.96%), anxious when receiving messages and emails (19.27%), socially isolated (14.51%), and suicidal (1.21%).

There were a few measurement tools for cyberbullying has been developed. The measurement tools were multi-factors and single factors. These measurement tools included the Cyber Victim and Bully Scale (CVBS) with three factor and 22 items [17], Cyberbullying Experience Survey (CES) with two factors and 48 items [18], Cyberbullying Questionnaire (CBQ) with two factors and 27 items [19], and Cyberbullying Scale (CBS) with one factor and 16 items [20]. A systematic assessment of cyberbullying is thus crucial for determining and detecting the events of cyberbullying due to its impact and severity. Several validated instruments have been developed to assess cyberbullying worldwide. However, none of them had been validated in the Malay version. 

Among those questionnaires related to Cyberbullying, the CBS consists of smallest number of items and required less time to complete. This one-factor cyberbullying victimization instrument was developed by Stewart et al. [20]. The scale comprises 16 items (two multiple-choice questions and 14 items rated on a Likert-type scale). This scale measures cyber victimization among teenagers and had been tested in English language. The results had shown good reliability and validity with the excellent internal consistency of Cronbach’s alpha 0.94. By considering the number of items and good criteria, CBS was selected in this study. 

The CBS has not been translated and validated for use in the context of adolescents in Malaysia. Therefore, the present study emphasizes the validation of the Malay version of the CBS (CBS-M), as well as looking into the correlation with depression, stress and anxiety (DASS-21). We hypothesized that there was a significant correlation between cyberbullying and psychological wellbeing among adolescence. In the present study the DASS-21 Malay version was used to measure the psychological wellbeing of the participants. If there is a significant correlation between CBS-M score with the depression, anxiety and stress from DASS-21, it would further support the validity of the CBS-M. 

## 2. Materials and Methods

### 2.1. Study Design, Participants

The study employed a cross-sectional study design. The study participants included secondary schools’ students around Muar, Johor, Malaysia who eligible and consented to join the study. The mean age of the exploratory factor analysis (EFA) participants was 14.7 (SD = 1.26) and that of confirmatory factor analysis (CFA) participants was 14.6 (SD = 1.25) years.

### 2.2. Instruments

Demographic Information. The questionnaire included items related to participants’ demographic characteristics (e.g., age, gender, and ethnicity, religion, number of siblings, parent’s income and education level).

Cyberbullying Scale (CBS). The CBS was developed by Stewart et al. [20]. It is a broad self-report measure of cyberbullying. The CBS comprises 14 questions rated using a five-point Likert-type scale (0 = Never, 1 = Almost Never, 2 = Sometimes, 3 = Almost All the Time, and 4 = All the Time). In terms of the validity and reliability of CBS for EFA, single factor loadings for individual items range from 0.72 to 0.90 with comparative fit index (CFI)/Tucker–Lewis index (TLI) = 0.98 and root-mean-square error of approximation (RMSEA) = 0.06.

Depression, Anxiety, and Stress Scale (DASS-21). Ramli et al. [21] translated and validated the Malay version of the Depression, Anxiety, and Stress Scale (DASS-21). The questionnaire comprises three domains, namely, depression, anxiety, and stress. Items were rated using a five-point Likert-type scale ranging from 1 = never to 4 = almost always. DASS-21 is the simplified version of DASS-41 [22], where items were reduced to 21 with seven items for each domain. The validation and reliability process of the translation obtained positive results in the Malaysian population with Cronbach’s alpha values of 0.84, 0.74, and 0.79 for depression, anxiety, and stress, respectively. It has significant factor loading values ranging from 0.39 to 0.73 for the majority of items. Correlations among the scales reached 0.54 to 0.68. The level of severity for each domain was determined based on scores (i.e., normal, mild, moderate, severe, and extremely severe).

### 2.3. Questionnaire Translation 

The forward and backward translation procedure was used in translating the English version of CBS into Malay language. Two bilingual translators were involved for forward translations (English to Malay) and another two bilingual translators were involved in backward translation (Malay to English). Both versions were compared, and preliminary version of CBS-M was established. Then, two experts with knowledge on questionnaire design and a clinical psychologist reviewed the CBS-M to ensure the content were culturally appropriate to the Malaysian population. The final version of CBS-M was pre-tested among 30 students to assess the clarity of the CBS-M. The CBS-M questionnaire is provided as supplemental material. 

### 2.4. Procedure

Ethical approval from the Human Research Ethics Committee of Universiti Sains Malaysia (USM/JEPeM/19080507), the National Medical Research Register (NMRR-19-2627-50601-(IIR)), and the Ministry of Education Malaysia (KPM.600-3/2/3-eras(4994)) was obtained. The study was conducted in accordance with the Declaration of Helsinki. 

The study was conducted in secondary schools in Muar, Johor, Malaysia in two phases, namely, exploratory and confirmatory. Multi-stage sampling was applied for sampling method. The study applied simple random sampling to select four out of 17 secondary schools. Thirty students from one of the four schools were randomly selected for pre-testing of the questionnaire. The school was excluded for the next sampling of EFA and CFA. The inclusion criteria were students who had experience using the Internet and Malaysian citizens and the exclusions criteria were special need students such as deaf, blind and slow learners and illiterate students.

Three other schools were selected for exploratory factor analysis (EFA) and confirmatory factor analysis (CFA). The subjects were students from the three secondary schools who provided written consent to participate as well as obtained permission from parents. Simple random sampling was used in classes. All participating students in the selected classes were further screened according to the inclusion (Malaysian students using the Internet) and exclusion (students with special needs, such as deaf, blind, slow learners, and illiterate) criteria. Parents’ written consent was obtained before inclusion in the study. The present study used the self-reported CBS-M and DASS-21. The participants voluntarily completed the CBS-M questionnaire and returned it to the researchers. The estimated time to complete the CBS-M was 10–15 min.

A total of 200 and 375 self-administered questionnaires were distributed, but only 138 and 263 students completed the questionnaire in the EFA and CFA phases, respectively.

### 2.5. Data Analysis

RStudio Version 1.2.5033 [23] was used to run exploratory factor analysis (EFA). Data in SPSS (IBM, Armonk, NY, USA) were imported into R by installing foreign package for the function of importing SPSS data. The packages foreign [24], psych [25] and lattice [26] were installed before the analysis can be run. Psych was used for psychometrics function and lattice was used for multivariate plots. All items of the CBS-M were individually checked through visual and statistical tests to assess univariate normality. The visual test for normality used a histogram and a statistical test (Shapiro–Wilk). The distributions of the items were non-normal, and the p-value for the Shapiro–Wilk test was significant (*p* < 0.05), which indicates that the distribution of data is non-normal. To verify multivariate normality, a kurtosis value of 27.92 and *p* < 0.05 were obtained, which exceeded the cutoff value of 5 [27]. Additionally, the dots on the Q–Q plot at the start and end deviated from the straight line. Therefore, factor extraction was conducted using principal axis factoring (PAF) as data were non-normally distributed. The model obtained a KMO value of 0.79. Therefore, it was acceptable for EFA. The Oblimin rotation method was used because the *p*-value of Bartlett’s test of sphericity is <0.05, which indicates a correlation among items. The number of factors was determined using Kaiser’s eigenvalues, Cattell’s scree test, parallel analysis, very simple structure (VSS), and Velicer’s minimum average partial (MAP). Kaiser’s eigenvalue rules out only constructs with eigenvalues of more than one, which should be retained for interpretation [28]. The eigenvalue can be interpreted as the amount of information in a factor. In other words, a cutoff value of one for eigenvalue indicates that the factor contains information for one item and is worthwhile to be extracted [29]. However, an eigenvalue less than one should not be retained because the factor contains information of less than one item or a single variable.

The scree plot [30] was used by determining the final substantial decline in the plot (elbow). The number of dots above the elbow of the plot is considered as the number of factors to be extracted. As the judgment of the scree plot can be subjective, researcher discretion is required [31]. Parallel analysis [32] is another method that can be used to determine the number of factors. A correlation matrix is computed from a randomly generated dataset that has the same numbers of observations and variables as the original data, and the eigenvalues are computed from the correlation matrix. The eigenvalues form randomly generated data, and original data are compared in the scree plot. The number of factors pertains to the number of points above the intersection of the plot. VSS is another alternative to determine the number of factors to be extracted from a correlation matrix [33]. VSS is produced by comparing the goodness-of-fit of the reduced (simple) structure matrix to the initial correlation matrix for a various number of factors (complexity 1, vss1). The highest value of VSS at complexity 1 (vss1) denotes the number of factors suggested for the model. Lastly, MAP is conducted by complete principal component analysis with a series of examinations of the average squared partial correlation, which is repeatedly computed [34]. The number of factors for the construct represents the step number as a result of the lowest average squared partial correlations [34].

Cronbach’s alpha coefficient was used to verify the internal consistency of the items for the CBS-M. A high Cronbach’s alpha value indicates high reliability. However, exceeding 0.95 indicates that many items are redundant. The corrected item-total correlation values with items < 0.5 can be considered for the omission, as suggested by Hair et al. [35].

Confirmatory factor analysis (CFA) was run for the CBS-M model after the EFA phase to confirm the measurement validity and reliability of the model. The packages used in RStudio software were foreign [24], psych [25] for psychometrics, lavaan [36] for CFA, semTools [37] for reliability and semPlot [38] for path diagram. The Mardia test for kurtosis was used to verify multivariate normality, and skewness was considered for *p*-value of <0.05, which indicates that the data were non-normally distributed. Therefore, the estimation method used was the robust maximum likelihood (MLR), which is a robust estimator with a robust standard error that yields the same parameter estimate, although the chi-square for the model test and standard errors for the parameters were calculated in a different manner, which rendered MLR robust to chi-square and standard error [39]. Overall model fitness was inspected using several fit indices. Based on the one-factor structure and measurement model of the 14 items, the fit indices used and cutoff values are the CFI and TLI with a cutoff value of >0.95, RMSEA with a cutoff value of ≤0.07, close-fit (ClfitRMSEA) value of >0.05, and SRMR with a cutoff value of ≤0.08 [35].

Composite reliability was obtained based on the final CFA model of the CBS-M. The recommended value for composite reliability is >0.7 [40], which indicates that a positive convergent validity was achieved and that the items belong to the same factor and share a high proportion of variance.

Spearman correlation was conducted to measure the correlation between CBS-M score and the three factors of DASS-21 (i.e., depression, anxiety and stress). Significant correlation between the DASS-21’s factors with cyberbullying would further support the validity of the CBS-M.

## 3. Results

### 3.1. Participants

Table 1 provides the demographic data for the EFA and CFA phases. 

### 3.2. EFA and Internal Consistency

**Figure 1:**
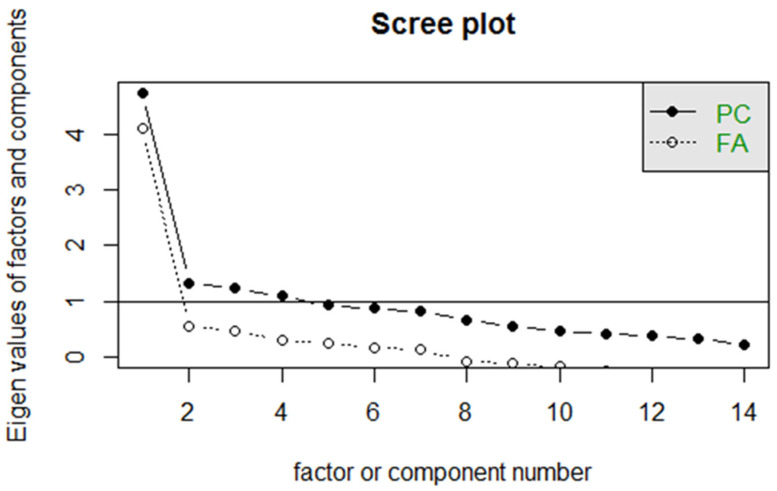
Scree plot of the Cyberbullying Scale-Malay (CBS-M) model. (PC: Principal Components, FA: Factor Analysis).

**Figure 2:**
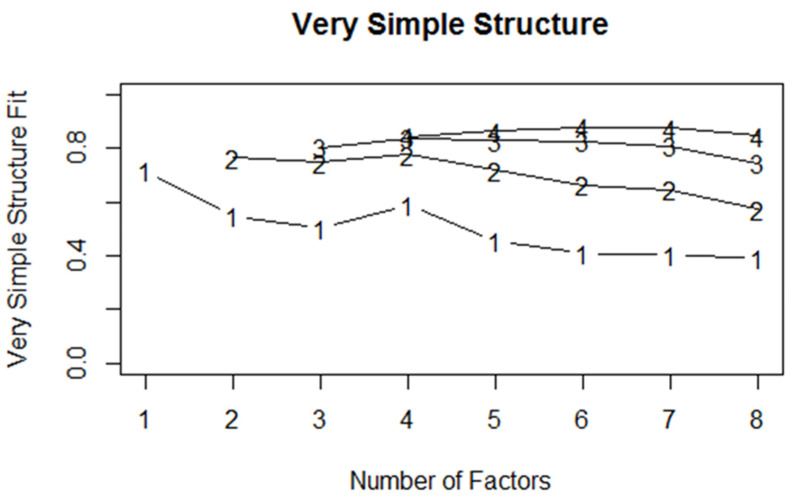
VSS plot of the CBS-M model.

Only one factor obtained an eigenvalue > 1 (4.11). The scree plot indicated that only one dot above the horizontal line of eigenvalues was equal to 1 (Figure 1). Figure 2 depicts the plot of vss1 with the number of factors. The vss1 for the CBS-M model was a result of achieving a maximum of 0.72 with one factor. MAP indicated the number of factors that minimize the MAP value. The model displays the result, which was achieved as a minimum value of 0.02 with one factor. This finding is similar to that for vss1. Therefore, the suggested factor for the model is one.

EFA analysis was run with the factor fitted as one using PAF and the Oblimin rotation method. The factor loadings ranged from 0.29 to 0.74. Table 2 presents the results of factor loadings and communalities. Although one item factor loading was <0.30, it was retained for the confirmatory phase. For internal consistency, the coefficient value of Cronbach’s alpha for the factor cyberbullying reached 0.87, which indicates a high level of reliability.

### 3.3. CFA and Composite Reliability

**Figure 3:**
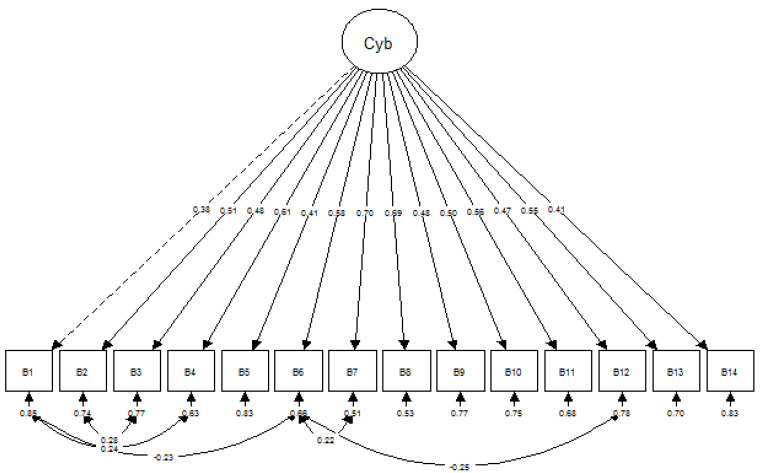
Final CBS-M model.

The initial measurement model of the CBS-M did not fit the data well. The fit indices lower than the acceptable recommended value for CFI and TLI (Table 3). The localized area of misfit, which was residuals, and MI were examined based on the CFA output. Several modifications were performed on the measurement model after the researcher obtained adequate theoretical support. The modification included adding the correlation between the residuals of the items. The final measurement model of CBS-M fit the data well based on several fit indices (Table 3). Composite reliability was obtained on the final measurement model of the CBS-M at 0.832, which indicates good reliability. The final measurement model retained all 14 items without omission and with cyberbullying as one factor. Figure 3 illustrates the final measurement model of the CBS-M.

### 3.4. Correlation between Cyberbullying and DASS-21’s Factors

The relationship between cyberbullying with stress, anxiety and depression was a positive linear relationship with fair correlations. The relationship of the correlations was shown in Table 4. The result had further supported the validity of CBS-M.

## 4. Discussion

Developing the CBS-M by translating and validating the CBS in the Malay language is important for measuring cyberbullying among adolescents in Malaysia. Stewart et al. [20] confirmed that the original version of CBS is a valid and reliable tool for use among adolescents in the United States. The present study obtained positive psychometric properties including positive correlations with the DASS-21 constructs (i.e., depression, anxiety and stress). In Malaysia, the level of awareness about cyberbullying is relatively low, and a tool for measuring cyberbullying in the Malay language is under-developed. As the Malay language and culture are unique and different, examining the psychometric properties of the Malay version of the CBS is important. The results could be beneficial in developing a cyberbullying tool for use among adolescents in Malaysia, thus increasing awareness regarding cyberbullying in the Malaysian context.

As previously mentioned, the original version of the CBS comprises 16 items. Out of the total items, 14 were rated using a Likert-type scale and thus subjected to EFA to assess the measurement validity of the model. To this end, the number of factors that can be extracted was determined. Additionally, the quality of the items under this model was explored and investigated after translation into the Malay language. The original version of the CBS was constructed with one factor, namely, cyberbullying victimization. In the present study, the initial eigenvalues and Cattel’s scree plot demonstrated that one factor obtained >1 above eigenvalue with a scree plot showing one dot above the horizontal line of the eigenvalues. Therefore, the factor number was fitted with one, as recommended by Stewart et al. [20]. The results of the factor loadings were examined, where one item was found to be slightly <0.3 (item B14: “How often did another kid pretend to be you and sent or posted something that damaged your reputation or friendships?”). The item was not omitted as it was considered important for measuring cyberbullying. Asking help from an adult or reporting to an adult was found as one of the actions that adolescents refuse to take when they become cyber victims [16,41,42].

Other items with low factor loadings were B1 (0.383; “How often do you get online or text messages from another kid threatening to beat you up or hurt you physically”), B5 (0.388; “How often do you get a text or online messages that make you afraid for your safety?”), and B9 (0.307; “How often does another kid send you a message saying they will beat you up if you do not do what they want you to do?”). These items mainly pertained to receiving threats on physical damage or safety, which were considered similar. Discussion with the clinical psychologist led to the consensus that the model should retain the three items because they measured important aspects of cyberbullying among adolescents. Communalities displayed a positive range from 0.0864 to 0.5564. No items were omitted from the model. The final EFA model confirmed that the model was a one-factor model, which consisted of the abovementioned 14 items. Additionally, the CBS-M displayed positive internal consistency with high Cronbach’s alpha values although less than those of the original CBS. The coefficient value of Cronbach’s alpha for cyberbullying as a factor was 0.87, which indicates a high level of internal consistency. All items were considered important for the measurement of cyberbullying. Therefore, no items were omitted, and the study proceeded to the CFA phase.

CFA was performed to confirm if the model of the 14 items fit the data well. The result was found to be similar to that of the original study using a single structure. In the present study, the MLR was used as a suitable estimator because of the non-normal distribution at the multivariate level. MLR is a robust estimator with a robust standard error that yields the same parameter estimates, although the chi-square for the model test and standard errors for the parameters were calculated differently, which rendered MLR robust to chi-square and standard error [40]. Overall model fitness was examined using overall fit-by-fit indices, localized area of misfit, and parameter estimates for model fitness. Analysis indicated that the CBS-M model was confirmed with one factor and 14 items. Composite reliability was computed after obtaining the final model. All factors demonstrated positive composite reliability with a CR value of 0.832.

The study has several limitations. The total number of respondents obtained was less than the calculated sample size, which may lead to skewness in the variables and influence the results. The respondents were recruited from schools, thus limiting sampling to school-goers alone. Thus, the results may not be representative of the total population of adolescents in Malaysia. The time of the test administration was less than the ideal time. The researcher could only approach the students when school ended at mid-day, which was between 1–2.30 pm. This was not the most effective time for the students to complete the questionnaire because their classes had ended and were expected to go home at the afternoon. Consequently, some students may hurriedly filled the form as they wanted to go home, which led to several missing data and incomplete answers. The other limitation was a correlation with another established questionnaires of cyberbullying. The researchers tested the correlation between CBS-M score with DASS-21 because DASS-21 has already translated into the Malay language and proven to be valid and reliable in Malaysia population. The study could be better if the CBS-M score can be correlated with another cyberbullying scale such as CBQ or CES. However, no other cyberbullying scale available in Malay and due to time constraint, the researchers suggest more cyberbullying scales should be translated and validated in future study. Another limitation was the risk factors, platforms and effects of cyberbullying among the adolescent were not studied. In Malaysia, the risk factors of cyberbully are still unknown. Effects of cyberbullying on school performance were also not investigated. Platforms of cyberbullying among the adolescents were also not included in the study.

The study recommends a replication on a larger, more representative sampling from different localities and socio-demographic backgrounds. Factors and elements related to cyberbullying, such as its risks for and effects on victims, perpetrators, and mechanism profiling were potential research areas in which the instrument can be used. The findings and information from such studies can benefit policymakers in formulating strategies to better prevent cyberbullying particularly among adolescents in Malaysia.

## 5. Conclusions

The study provided a valid and reliable instrument for measuring cyberbullying in the Malay language. The final CFA model indicated a good fit and a valid and reliable model structure without the omission of items. Therefore, the CBS-M model is a valid and reliable tool for measuring cyberbullying among adolescents in Malaysia.

## Figures and Tables

**Table 1:** Sociodemographic data of participants for EFA (*n* = 138) and CFA (*n* = 263).

Variables	EFA	CFA	Total
	Frequency (%)	Frequency (%)	
Age	14.7 (1.26) *	14.6 (1.25) *	14.61 (1.25) *
Gender			
Male	47 (34.1)	88 (33.5)	135 (33.7)
Female	91 (65.9)	174 (66.2)	265 (66.1)
Missing	–	1 (0.4)	1 (0.2)
Number of siblings	3.99 (1.47) *	3.96 (1.51) *	3.97 (1.49)
Ethnicity			
Malay	124 (89.9)	226 (85.9)	350 (87.3)
Chinese	14 (10.1)	35 (13.3)	49 (12.2)
Indian	–	2 (0.8)	2 (0.5)
Others	–	–	–
Religion			
Islam	124 (89.9)	226 (85.9)	350 (87.3)
Buddha	14 (10.1)	34 (12.9)	48 (12.0)
Hindu	–	1 (0.4)	1 (0.2)
Christian	–	1 (0.4)	1 (0.2)
Missing	–	1 (0.4)	1 (0.2)
Father’s income			
None	3 (2.2)	5 (1.9)	8 (2.0)
<3000	57 (41.3)	117 (44.5)	174 (43.4)
3001–6000	11 (8.0)	29 (11.0)	40 (10.0)
6001–12,999	11 (8.0)	14 (5.3)	25 (6.2)
>13,000	3 (2.2)	6 (2.3)	9 (2.2)
Unknown	53 (38.4)	92 (35.0)	145 (36.2)
Mother’s income			
None	45 (32.6)	89 (33.8)	134 (33.4)
<3000	27 (19.6)	63 (24.0)	90 (22.4)
3001–6000	13 (9.4)	28 (10.6)	41 (10.2)
6001–12,999	12 (8.7)	14 (5.3)	26 (6.5)
>13,000	4 (2.9)	2 (0.8)	6 (1.5)
Unknown	37 (26.8)	67 (25.5)	104 (25.9)
Father’s educational level			
None	3 (2.2)	3 (1.1)	6 (1.5)
Primary education	1 (0.7)	13 (4.9)	14 (3.5)
Secondary education	53 (38.4)	97 (36.9)	150 (37.4)
Post-secondary education	5 (3.6)	12 (4.6)	17 (4.2)
Tertiary education	42 (30.4)	67 (25.5)	109 (27.2)
Unknown	34 (24.6)	71 (27.0)	105(26.2)
Mother’s educational level			
None	1 (0.7)	2 (0.8)	3 (0.7)
Primary education	5 (3.6)	9 (3.4)	14 (3.5)
Secondary education	49 (35.5)	97 (36.9)	146 (36.4)
Post-secondary education	3 (2.2)	8 (3.0)	11 (2.7)
Tertiary education	51 (37.0)	84 (31.9)	135 (33.7)
Unknown	29 (21.0)	63 (24.0)	92 (22.9)

Note. * Mean (SD).

**Table 2:** Factor loadings, communalities, and item complexities of the CBS-M with the one-factor model.

Factor	Item	Factor Loading	Communalities (Extraction)
Cyberbullying	B1	0.383	0.1471
	B2	0.624	0.3900
	B3	0.544	0.2965
	B4	0.579	0.3354
	B5	0.388	0.1508
	B6	0.746	0.5564
	B7	0.617	0.3803
	B8	0.640	0.4090
	B9	0.307	0.0941
	B10	0.489	0.2387
	B11	0.713	0.5086
	B12	0.406	0.1651
	B13	0.592	0.3502
	B14	0.294	0.0864

Note. KMO value = 0.79; Bartlett’s test of sphericity was significant (*p* < 0.001). Principal axis factoring was applied. Rotation method: Oblimin method.

**Table 3:** Comparison of fit indices among the CBS-M models.

Model	χ^2^ (df)	*p*-Value	SRMR	RMSEA	90% CI	CFI	TLI	AIC	BIC
Model 1	154.2 (77)	0.001	0.066	0.077	0.059, 0.095	0.858	0.832	8365	8465
Model 2	139.7 (76)	0.001	0.064	0.071	0.052, 0.089	0.883	0.859	8345	8448
Model 3	129.2 (75)	0.001	0.061	0.065	0.046, 0.084	0.900	0.879	8329	8436
Model 4	121.0 (74)	0.001	0.059	0.061	0.041, 0.080	0.913	0.893	8318	8429
Model 5	111.2 (73)	0.003	0.057	0.056	0.033, 0.076	0.930	0.912	8306	8420
Model 6 (final model)	101.3 (72)	0.013	0.055	0.049	0.024, 0.071	0.946	0.932	8294	8411

Note. Model 2: Residual correlations was added on items B6 and B7, Model 3: Residual correlations was added on items B2 and B3, Model 4: Residual correlations was added on items B1 and B4, Model 5: Residual correlations was added on items B6 and B12, Model 6: Residual correlations was added on items B1 and B6.

**Table 4:** Correlation between CBS-M and DASS-21 factors (*n* = 401).

DASS-21 Factors	r *	*p*-Value
	Cyberbullying	
Stress	0.44	0.001
Anxiety	0.41	0.001
Depression	0.40	0.001

Note. * r = correlation coefficient.

## Data Availability

The data is available upon request from the authors.

## Supplementary Materials

The following are available online at https://www.mdpi.com/article/10.3390/ijerph182111669/s1, Material 1: Skala Buli-siber [Cyberbullying Scale (CBS)].
