# Supplementary material for: The Validity and Reliability of the Malay Version of the Cyberbullying Scale among Secondary School Adolescents in Malaysia
Source: Int J Environ Res Public Health. 2021 Nov 6;18(21):11669. doi: 10.3390/ijerph182111669 (PMC8582711; doi:10.3390/ijerph182111669)
Supplement: Supplementary file 1 [file ijerph-18-11669-s001.zip › ijerph-1389826-supplementary.pdf]

Skala Buli-siber [*Cyberbullying Scale (CBS)*]

Soalan berikut adalah berkenaan apa yang berlaku dalam hidup anda dalam beberapa bulan yang lepas. Sila bulatkan jawapan yang terbaik.

1. Adakah kanak-kanak lain menggunakan mana-mana yang berikut untuk membuli anda? (Bulatkan semua yang pernah terjadi kepada anda)

a) Emel
b) Mesej teks
c) Mesej bergambar
d) Mesej segera contoh whatsapp
e) Membina laman sesawang atau kumpulan mesej untuk anda
f) Klip video dalam talian berkenaan anda
g) Tapak rangkaian sosial (Facebook)
h) Bilik sembang
i) Dunia maya (seperti Second Life atau Sims)

2. Adakah anda menggunakan mana-mana yang berikut untuk membuli kanak-kanak lain? (bulatkan semua yang anda telah gunakan untuk membuli)

a) Emel
b) Mesej teks
c) Mesej Bergambar
d) Mesej segera contoh whatsapp
e) Membina laman sesawang atau kumpulan mesej untuk anda
f) Klip video dalam talian berkenaan anda
g) Tapak rangkaian sosial (seperti Facebook)
h) Bilik sembang
i) Dunia maya (seperti Second Life atau Sims)

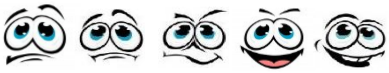

Sila jawab soalan-soalan berikut:	1.Tidak Pernah	2. Hampir tidak pernah	3.Kadang-kadang	4.Hampir sepanjang masa	5.Sepanjang masa
1. Menerima ugutan melalui dalam talian atau mesej teks daripada kanak-kanak lain untuk memukul atau mencederakan anda	1	2	3	4	5
2. Kanak-kanak lain secara sengaja mengabaikan anda dalam kumpulan atas talian	1	2	3	4	5
3. Kanak-kanak lain mempersendakan anda (seperti memanggil nama anda dengan nama panggilan yang tidak sepatutnya atau mentertawa anda) dalam mesej teks atau talian	1	2	3	4	5
4. Kanak-kanak yang marah pada anda cuba membalas dendam dengan tidak membenarkan anda menyertai kumpulan atas talian mereka	1	2	3	4	5
5. Anda menerima mesej teks atau mesej atas talian yang membuatkan keselamatan anda tergugat	1	2	3	4	5
6. Seorang kanak-kanak membuat cerita palsu tentang anda dan membuatkan kawan-kawan lain menjauhi anda	1	2	3	4	5
7. Seorang kanak-kanak anda mengatakan mereka akan menjauhi anda selagi anda tidak menuruti kehendak mereka	1	2	3	4	5
8. Kanak-kanak lain anda cuba menghasut rakan-rakan yang lain untuk tidak berkawan dengan anda dengan menghantar mesej dan memuat naik paparan yang tidak sepatutnya tentang anda	1	2	3	4	5
9. Kanak-kanak lain menghantar mesej mengatakan mereka akan memukul anda jika anda tidak menurut kehendak mereka	1	2	3	4	5
10. Anda terlibat dengan pergaduhan atas talian	1	2	3	4	5
11. Kanak-kanak lain memuat naik paparan yang menjejaskan reputasi atau persahabatan anda hingga menyebabkan emosi anda terganggu	1	2	3	4	5
12. Kanak-kanak lain mencuri identiti anda dan memuat naik paparan yang menjejaskan reputasi atau persahabatan anda dengan kawan-kawan yang lain	1	2	3	4	5
13. Kanak-kanak lain berkongsi rahsia atau gambar peribadi anda tanpa kebenaran	1	2	3	4	5
14. Anda terpaksa meminta bantuan daripada orang dewasa untuk menyelesaikan masalah yang terjadi atas talian	1	2	3	4	5
